# Ethyl 4-(3-chloro­phen­yl)-3,6-dihydr­oxy-6-methyl-2-(2-pyrid­yl)-4,5,6,7-tetra­hydro­indazole-5-carboxyl­ate

**DOI:** 10.1107/S1600536810009360

**Published:** 2010-03-17

**Authors:** S. Amirthaganesan, G. Aridoss, D.Gayathri, Keun Soo Park, Yeon Tae Jeong

**Affiliations:** a Division of Image Science and Information Engineering, Pukyong National University, Busan 608-739, Republic of Korea; b Institute of Structural Biology and Biophysics-2: Molecular Biophysics, Research Centre Jülich, D-52425 Jülich, Germany

## Abstract

In the title compound, C_22_H_22_ClN_3_O_4_, the cyclo­hexane ring adopts a twisted half-chair conformation. The mol­ecule is stabilized by an intra­molecular O—H⋯N inter­action, generating an *S*(6) motif. The crystal packing is stabilized by inter­molecular O—H⋯N and C—H⋯O inter­actions.

## Related literature

For the synthesis and stereochemistry investigations through NMR of *N*(2)-pyridyl tetra­hydro­indazoles, see: Amirthaganesan *et al.* (2008 ▶). For the biological activity of tetra­hydro­indazoles, see: Connolly *et al.* (1997 ▶). For ring conformational analysis, see: Cremer & Pople (1975 ▶); Nardelli (1983 ▶).
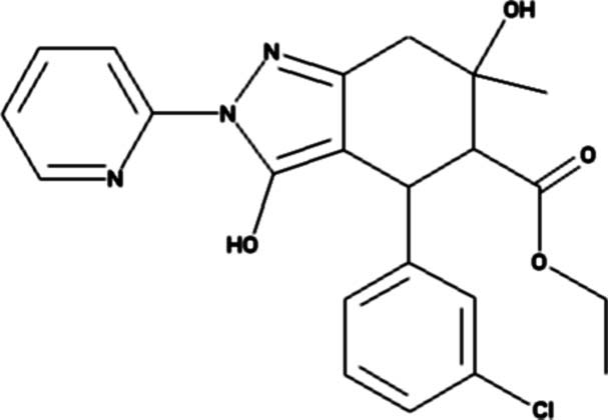


## Experimental

### 

#### Crystal data

C_22_H_22_ClN_3_O_4_
*M* *_r_* = 427.88
Triclinic, 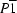

*a* = 8.585 (5) Å
*b* = 9.053 (3) Å
*c* = 14.884 (3) Å
α = 94.68 (2)°
β = 90.19 (2)°
γ = 115.66 (3)°
*V* = 1038.3 (7) Å^3^
*Z* = 2
Mo *K*α radiation
μ = 0.22 mm^−1^
*T* = 293 K
0.30 × 0.22 × 0.20 mm

#### Data collection

Bruker Kappa APEXII CCD diffractometer
Absorption correction: multi-scan (*SADABS*; Bruker, 2004 ▶) *T*_min_ = 0.937, *T*_max_ = 0.958
4446 measured reflections
3647 independent reflections
3025 reflections with *I* > 2σ(*I*)
*R*_int_ = 0.010

#### Refinement

*R*[*F* ^2^ > 2σ(*F* ^2^)] = 0.033
*wR*(*F* ^2^) = 0.092
*S* = 1.04
3647 reflections
274 parameters
H-atom parameters constrained
Δρ_max_ = 0.24 e Å^−3^
Δρ_min_ = −0.30 e Å^−3^

### 

Data collection: *APEX2* (Bruker, 2004 ▶); cell refinement: *SAINT* (Bruker, 2004 ▶); data reduction: *SAINT*; program(s) used to solve structure: *SIR92* (Altomare *et al.*, 1999 ▶); program(s) used to refine structure: *SHELXL97* (Sheldrick, 2008 ▶); molecular graphics: *PLATON* (Spek, 2009 ▶); software used to prepare material for publication: *SHELXL97*.

## Supplementary Material

Crystal structure: contains datablocks I, global. DOI: 10.1107/S1600536810009360/ng2744sup1.cif

Structure factors: contains datablocks I. DOI: 10.1107/S1600536810009360/ng2744Isup2.hkl

Additional supplementary materials:  crystallographic information; 3D view; checkCIF report

## Figures and Tables

**Table 1:** Hydrogen-bond geometry (Å, °)

*D*—H⋯*A*	*D*—H	H⋯*A*	*D*⋯*A*	*D*—H⋯*A*
O4—H4⋯N3	0.82	1.90	2.604 (3)	143
O3—H3⋯N1^i^	0.82	2.10	2.920 (2)	176
C11—H11⋯O3^ii^	0.93	2.58	3.418 (3)	151
C16—H16*C*⋯O4^iii^	0.96	2.57	3.397 (3)	144

Symmetry codes: (i) 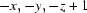; (ii) 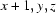; (iii) 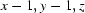.

**Fig. 1.**
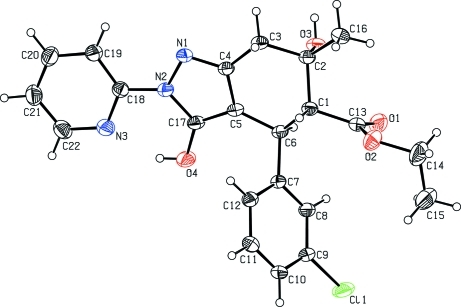
The molecular structure of title compound, showing 30% probability displacement ellipsoids.

**Fig. 2.**
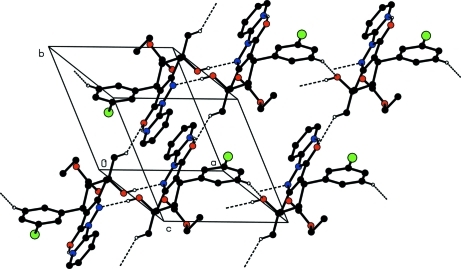
The molecular packing of (I). For clarity, hydrogen atoms which are not involved in hydrogen bonding are omitted.

## supplementary crystallographic information

### Comment

In azole family, tetrahydroindazoles (cycloalkane derivatives of pyrazoles) are having much importance for their effective biological potencies (Connolly *et al.*, 1997). Our current research work is focused on the stereospecific synthesis of 1(H) and various N-substituted tetrahydroindazoles by taking cyclic β keto esters as an effective synthons, and exploring their stereochemistry. Recently, we have described complete structural elucidation and conformation of a series of N(2)-pyridyl tetrahydroindazoles (Amirthaganesan *et al.*, 2008). One and two dimensional NMR investigations strongly proved that all the compounds obtained as a single isomer where cyclohexane ring adopts slightly distorted chair conformation and pyridyl moiety favored at N(2) position in the azole ring. We report here the X-ray crystal structure of the title compound.

The sum of the bond angles at N2 (359.9 (3)°) indicates the sp^2^ hybridization. Atoms O4 and Cl1 lie in the plane of the rings to which they are attached with the deviation of 0.002 (2) and -0.004 (1) Å, respectively. The pyridine (or pyridyl) ring, attached at N(2) position of the pyrazole ring, is parallel to the pyrazole ring with the dihedral angle of 8.0 (1)°. The dihedral angle between the phenyl ring and the pyridine (or pyridyl) ring is 64.2 (1)°. Torsion angle (3.9 (3)°) around O1—C13—O2—C14 indicates the planarity of the moiety. The cyclohexane ring adopts twisted half-chair conformation in solid state, with the puckering parameters (Cremer & Pople, 1975) and the smallest displacement asymmetry parameters (Nardelli, 1983) being q_2_ = 0.373 (2) Å, q_3_ = -0.346 (2) Å; Q_T_ = 0.509 (2)Å and θ = 132.8 (2)°.

The molecule is stabilized by strong O—H···N intramolecular interaction, wherein, atom O4 acts as donor to N3 generating S(6) motif. The crystal packing is stabilized by O—H···N and C—H···O intermolecular interactions. Atoms C11 and C16 act as donors to O3 and O4, respectively, each generating chain of C(8), which in turn generates R_4_^4^(28) graph set along ab plane. Atom O3 acts as donor to N1 at (-x,-y,-z+1) generating a centrosymmetric dimer of R_2_^2^(12) graph set.

### Experimental

A mixture of r-2,c(4)-bis(ethoxycarbonyl)-c(5)-hydroxy-t(5)-methyl-t(3)- (*p*-chlorophenyl)cyclohexanone (1 mmol) and 2-hydrazinopyridine (1.2 mmol) in toluene with the addition of catalytic amount of acetic acid were refluxed for about 6 h. After completion of the reaction, the solvent was evaporated under vacuum and the resultant residue was recrystallized from ethanol.

### Refinement

All H-atoms were refined using a riding model with d(C—H) = 0.93 Å, U_iso_ = 1.2U_eq_ (C) for aromatic, 0.98 Å, U_iso_ = 1.2U_eq_ (C) for CH, 0.97 Å, U_iso_ = 1.2U_eq_ (C) for CH_2_ 0.96 Å, U_iso_ = 1.5U_eq_ (C) for CH_3_ atoms and d(O—H) = 0.82 Å, U_iso_(H) = 1.5U_eq_ (O) for the OH group.

### Crystal data

**Table:**

C_22_H_22_ClN_3_O_4_	*Z* = 2
*M**_r_* = 427.88	*F*(000) = 448
Triclinic, *P*1	*D*_x_ = 1.369 Mg m^−^^3^
Hall symbol: -P 1	Mo *K*α radiation, λ = 0.71073 Å
*a* = 8.585 (5) Å	Cell parameters from 1965 reflections
*b* = 9.053 (3) Å	θ = 2.5–25.0°
*c* = 14.884 (3) Å	µ = 0.22 mm^−^^1^
α = 94.68 (2)°	*T* = 293 K
β = 90.19 (2)°	Prism, colourless
γ = 115.66 (3)°	0.30 × 0.22 × 0.20 mm
*V* = 1038.3 (7) Å^3^	

### Data collection

**Table:**

Bruker Kappa APEXII CCD diffractometer	3647 independent reflections
Radiation source: fine-focus sealed tube	3025 reflections with *I* > 2σ(*I*)
graphite	*R*_int_ = 0.010
ω and φ scan	θ_max_ = 25.0°, θ_min_ = 2.5°
Absorption correction: multi-scan (*SADABS*; Bruker, 2004)	*h* = −1→10
*T*_min_ = 0.937, *T*_max_ = 0.958	*k* = −10→10
4446 measured reflections	*l* = −17→17

### Refinement

**Table:**

Refinement on *F*^2^	Secondary atom site location: difference Fourier map
Least-squares matrix: full	Hydrogen site location: inferred from neighbouring sites
*R*[*F*^2^ > 2σ(*F*^2^)] = 0.033	H-atom parameters constrained
*wR*(*F*^2^) = 0.092	*w* = 1/[σ^2^(*F*_o_^2^) + (0.0426*P*)^2^ + 0.3338*P*] where *P* = (*F*_o_^2^ + 2*F*_c_^2^)/3
*S* = 1.04	(Δ/σ)_max_ < 0.001
3647 reflections	Δρ_max_ = 0.24 e Å^−^^3^
274 parameters	Δρ_min_ = −0.30 e Å^−^^3^
0 restraints	Extinction correction: *SHELXL97* (Sheldrick, 2008), Fc^*^=kFc[1+0.001xFc^2^λ^3^/sin(2θ)]^-1/4^
Primary atom site location: structure-invariant direct methods	Extinction coefficient: 0.0065 (15)

### Special details

**Table:**

Geometry. All esds (except the esd in the dihedral angle between two l.s. planes) are estimated using the full covariance matrix. The cell esds are taken into account individually in the estimation of esds in distances, angles and torsion angles; correlations between esds in cell parameters are only used when they are defined by crystal symmetry. An approximate (isotropic) treatment of cell esds is used for estimating esds involving l.s. planes.
Refinement. Refinement of *F*^2^ against ALL reflections. The weighted *R*-factor wR and goodness of fit *S* are based on *F*^2^, conventional *R*-factors *R* are based on *F*, with *F* set to zero for negative *F*^2^. The threshold expression of *F*^2^ > σ(*F*^2^) is used only for calculating *R*-factors(gt) etc. and is not relevant to the choice of reflections for refinement. *R*-factors based on *F*^2^ are statistically about twice as large as those based on *F*, and *R*- factors based on ALL data will be even larger.

### Fractional atomic coordinates and isotropic or equivalent isotropic displacement parameters (Å^2^)

**Table:**

	*x*	*y*	*z*	*U*_iso_*/*U*_eq_	
C1	0.23589 (18)	0.01801 (18)	0.72716 (10)	0.0366 (3)	
H1	0.2978	−0.0511	0.7220	0.044*	
C2	0.07219 (19)	−0.06547 (18)	0.66275 (10)	0.0375 (3)	
C3	0.12780 (19)	−0.09587 (18)	0.56754 (10)	0.0396 (4)	
H3A	0.1634	−0.1841	0.5665	0.048*	
H3B	0.0304	−0.1297	0.5248	0.048*	
C4	0.27363 (19)	0.05594 (18)	0.54012 (10)	0.0361 (3)	
C5	0.38310 (19)	0.18470 (18)	0.60252 (10)	0.0369 (3)	
C6	0.36178 (19)	0.19180 (18)	0.70250 (10)	0.0356 (3)	
H6	0.3088	0.2666	0.7180	0.043*	
C7	0.53145 (18)	0.25391 (18)	0.75799 (10)	0.0351 (3)	
C8	0.5566 (2)	0.3545 (2)	0.83731 (10)	0.0414 (4)	
H8	0.4731	0.3891	0.8551	0.050*	
C9	0.7067 (2)	0.4029 (2)	0.88964 (10)	0.0447 (4)	
C10	0.8331 (2)	0.3542 (2)	0.86565 (11)	0.0469 (4)	
H10	0.9332	0.3878	0.9019	0.056*	
C11	0.8082 (2)	0.2543 (2)	0.78656 (11)	0.0469 (4)	
H11	0.8920	0.2196	0.7693	0.056*	
C12	0.6592 (2)	0.2054 (2)	0.73290 (11)	0.0417 (4)	
H12	0.6445	0.1393	0.6794	0.050*	
C13	0.1873 (2)	0.0274 (2)	0.82426 (11)	0.0427 (4)	
C14	0.1681 (4)	−0.0911 (3)	0.96324 (14)	0.0803 (7)	
H14A	0.1570	−0.1955	0.9816	0.096*	
H14B	0.0556	−0.0903	0.9672	0.096*	
C15	0.2938 (3)	0.0447 (3)	1.02645 (15)	0.0851 (7)	
H15A	0.4060	0.0462	1.0219	0.128*	
H15B	0.2568	0.0276	1.0871	0.128*	
H15C	0.2997	0.1479	1.0111	0.128*	
C16	−0.0561 (2)	−0.2271 (2)	0.69547 (12)	0.0507 (4)	
H16A	−0.0970	−0.2058	0.7528	0.076*	
H16B	−0.0002	−0.2976	0.7018	0.076*	
H16C	−0.1521	−0.2799	0.6525	0.076*	
C17	0.5006 (2)	0.29568 (19)	0.55071 (10)	0.0413 (4)	
C18	0.5488 (2)	0.30146 (19)	0.38582 (11)	0.0402 (4)	
C19	0.4867 (2)	0.2333 (2)	0.29936 (11)	0.0466 (4)	
H19	0.3820	0.1401	0.2887	0.056*	
C20	0.5862 (3)	0.3088 (2)	0.22937 (12)	0.0575 (5)	
H20	0.5490	0.2663	0.1701	0.069*	
C21	0.7400 (3)	0.4464 (3)	0.24685 (13)	0.0605 (5)	
H21	0.8084	0.4975	0.2000	0.073*	
C22	0.7904 (2)	0.5064 (2)	0.33443 (13)	0.0584 (5)	
H22	0.8939	0.6006	0.3462	0.070*	
N1	0.31575 (16)	0.08157 (15)	0.45512 (8)	0.0394 (3)	
N2	0.45910 (16)	0.23300 (15)	0.46190 (8)	0.0408 (3)	
N3	0.69768 (19)	0.43596 (18)	0.40430 (10)	0.0510 (4)	
O1	0.12768 (17)	0.11693 (18)	0.85661 (8)	0.0608 (4)	
O2	0.21903 (18)	−0.07785 (16)	0.87015 (8)	0.0602 (3)	
O3	−0.00213 (13)	0.04846 (13)	0.66127 (7)	0.0443 (3)	
H3	−0.0884	0.0092	0.6272	0.066*	
O4	0.63299 (16)	0.44010 (14)	0.57502 (8)	0.0589 (3)	
H4	0.6822	0.4810	0.5300	0.088*	
Cl1	0.73648 (7)	0.52862 (8)	0.98989 (3)	0.0772 (2)	

### Atomic displacement parameters (Å^2^)

**Table:**

	*U*^11^	*U*^22^	*U*^33^	*U*^12^	*U*^13^	*U*^23^
C1	0.0313 (7)	0.0407 (8)	0.0381 (8)	0.0167 (7)	−0.0041 (6)	−0.0003 (6)
C2	0.0317 (7)	0.0382 (8)	0.0398 (8)	0.0136 (6)	−0.0052 (6)	−0.0027 (6)
C3	0.0354 (8)	0.0369 (8)	0.0406 (8)	0.0118 (7)	−0.0072 (6)	−0.0052 (6)
C4	0.0328 (7)	0.0377 (8)	0.0358 (8)	0.0147 (6)	−0.0055 (6)	−0.0031 (6)
C5	0.0352 (8)	0.0366 (8)	0.0348 (8)	0.0128 (6)	−0.0085 (6)	−0.0031 (6)
C6	0.0326 (7)	0.0380 (8)	0.0347 (8)	0.0153 (6)	−0.0073 (6)	−0.0039 (6)
C7	0.0314 (7)	0.0369 (8)	0.0327 (7)	0.0113 (6)	−0.0038 (6)	0.0006 (6)
C8	0.0338 (8)	0.0494 (9)	0.0390 (8)	0.0178 (7)	−0.0043 (6)	−0.0045 (7)
C9	0.0370 (8)	0.0543 (10)	0.0334 (8)	0.0126 (7)	−0.0061 (6)	−0.0048 (7)
C10	0.0304 (8)	0.0625 (11)	0.0408 (9)	0.0138 (8)	−0.0064 (6)	0.0052 (8)
C11	0.0347 (8)	0.0575 (10)	0.0494 (10)	0.0211 (8)	0.0011 (7)	0.0042 (8)
C12	0.0388 (8)	0.0449 (9)	0.0388 (8)	0.0171 (7)	−0.0017 (6)	−0.0036 (7)
C13	0.0309 (8)	0.0504 (9)	0.0407 (9)	0.0125 (7)	−0.0048 (6)	0.0017 (7)
C14	0.1040 (18)	0.0827 (15)	0.0467 (11)	0.0304 (14)	0.0103 (11)	0.0233 (11)
C15	0.0936 (17)	0.114 (2)	0.0490 (12)	0.0472 (16)	−0.0075 (11)	0.0030 (12)
C16	0.0395 (9)	0.0468 (9)	0.0552 (10)	0.0094 (8)	−0.0018 (8)	0.0015 (8)
C17	0.0393 (8)	0.0374 (8)	0.0394 (8)	0.0106 (7)	−0.0094 (7)	−0.0029 (6)
C18	0.0387 (8)	0.0418 (8)	0.0432 (9)	0.0201 (7)	−0.0002 (7)	0.0050 (7)
C19	0.0447 (9)	0.0489 (9)	0.0447 (9)	0.0197 (8)	−0.0006 (7)	0.0004 (7)
C20	0.0642 (12)	0.0695 (12)	0.0421 (10)	0.0326 (10)	0.0043 (8)	0.0030 (9)
C21	0.0576 (11)	0.0710 (13)	0.0536 (11)	0.0264 (10)	0.0143 (9)	0.0183 (10)
C22	0.0458 (10)	0.0565 (11)	0.0648 (12)	0.0127 (9)	0.0058 (9)	0.0166 (9)
N1	0.0339 (7)	0.0389 (7)	0.0380 (7)	0.0102 (6)	−0.0048 (5)	−0.0038 (5)
N2	0.0373 (7)	0.0400 (7)	0.0364 (7)	0.0093 (6)	−0.0040 (5)	0.0005 (5)
N3	0.0447 (8)	0.0494 (8)	0.0506 (8)	0.0123 (7)	−0.0010 (6)	0.0075 (7)
O1	0.0593 (8)	0.0877 (10)	0.0464 (7)	0.0437 (8)	0.0021 (6)	−0.0024 (6)
O2	0.0762 (9)	0.0632 (8)	0.0440 (7)	0.0315 (7)	0.0015 (6)	0.0137 (6)
O3	0.0353 (6)	0.0488 (6)	0.0494 (6)	0.0210 (5)	−0.0127 (5)	−0.0083 (5)
O4	0.0561 (7)	0.0446 (7)	0.0471 (7)	−0.0039 (6)	−0.0076 (6)	−0.0020 (5)
Cl1	0.0581 (3)	0.1098 (5)	0.0520 (3)	0.0334 (3)	−0.0211 (2)	−0.0374 (3)

### Geometric parameters (Å, °)

**Table:**

C1—C13	1.512 (2)	C13—O2	1.333 (2)
C1—C6	1.550 (2)	C14—O2	1.453 (2)
C1—C2	1.555 (2)	C14—C15	1.489 (3)
C1—H1	0.9800	C14—H14A	0.9700
C2—O3	1.4300 (19)	C14—H14B	0.9700
C2—C16	1.521 (2)	C15—H15A	0.9600
C2—C3	1.538 (2)	C15—H15B	0.9600
C3—C4	1.492 (2)	C15—H15C	0.9600
C3—H3A	0.9700	C16—H16A	0.9600
C3—H3B	0.9700	C16—H16B	0.9600
C4—N1	1.328 (2)	C16—H16C	0.9600
C4—C5	1.405 (2)	C17—O4	1.3287 (19)
C5—C17	1.369 (2)	C17—N2	1.377 (2)
C5—C6	1.500 (2)	C18—N3	1.337 (2)
C6—C7	1.525 (2)	C18—C19	1.379 (2)
C6—H6	0.9800	C18—N2	1.402 (2)
C7—C8	1.387 (2)	C19—C20	1.378 (3)
C7—C12	1.389 (2)	C19—H19	0.9300
C8—C9	1.381 (2)	C20—C21	1.372 (3)
C8—H8	0.9300	C20—H20	0.9300
C9—C10	1.375 (2)	C21—C22	1.363 (3)
C9—Cl1	1.7482 (17)	C21—H21	0.9300
C10—C11	1.381 (2)	C22—N3	1.340 (2)
C10—H10	0.9300	C22—H22	0.9300
C11—C12	1.384 (2)	N1—N2	1.3854 (19)
C11—H11	0.9300	O3—H3	0.8200
C12—H12	0.9300	O4—H4	0.8200
C13—O1	1.204 (2)		
			
C13—C1—C6	109.76 (12)	O1—C13—O2	123.97 (16)
C13—C1—C2	111.12 (12)	O1—C13—C1	125.16 (15)
C6—C1—C2	113.02 (12)	O2—C13—C1	110.87 (14)
C13—C1—H1	107.6	O2—C14—C15	112.8 (2)
C6—C1—H1	107.6	O2—C14—H14A	109.0
C2—C1—H1	107.6	C15—C14—H14A	109.0
O3—C2—C16	110.78 (13)	O2—C14—H14B	109.0
O3—C2—C3	109.40 (13)	C15—C14—H14B	109.0
C16—C2—C3	110.15 (13)	H14A—C14—H14B	107.8
O3—C2—C1	106.58 (12)	C14—C15—H15A	109.5
C16—C2—C1	111.06 (13)	C14—C15—H15B	109.5
C3—C2—C1	108.78 (12)	H15A—C15—H15B	109.5
C4—C3—C2	110.91 (12)	C14—C15—H15C	109.5
C4—C3—H3A	109.5	H15A—C15—H15C	109.5
C2—C3—H3A	109.5	H15B—C15—H15C	109.5
C4—C3—H3B	109.5	C2—C16—H16A	109.5
C2—C3—H3B	109.5	C2—C16—H16B	109.5
H3A—C3—H3B	108.0	H16A—C16—H16B	109.5
N1—C4—C5	113.24 (14)	C2—C16—H16C	109.5
N1—C4—C3	123.79 (13)	H16A—C16—H16C	109.5
C5—C4—C3	122.95 (14)	H16B—C16—H16C	109.5
C17—C5—C4	104.49 (13)	O4—C17—C5	130.01 (14)
C17—C5—C6	130.71 (13)	O4—C17—N2	122.42 (15)
C4—C5—C6	124.75 (14)	C5—C17—N2	107.56 (13)
C5—C6—C7	113.80 (13)	N3—C18—C19	123.45 (16)
C5—C6—C1	108.64 (12)	N3—C18—N2	114.62 (14)
C7—C6—C1	110.03 (12)	C19—C18—N2	121.93 (15)
C5—C6—H6	108.1	C20—C19—C18	117.28 (17)
C7—C6—H6	108.1	C20—C19—H19	121.4
C1—C6—H6	108.1	C18—C19—H19	121.4
C8—C7—C12	118.85 (14)	C21—C20—C19	120.22 (18)
C8—C7—C6	119.86 (13)	C21—C20—H20	119.9
C12—C7—C6	121.22 (13)	C19—C20—H20	119.9
C9—C8—C7	119.45 (15)	C22—C21—C20	118.54 (18)
C9—C8—H8	120.3	C22—C21—H21	120.7
C7—C8—H8	120.3	C20—C21—H21	120.7
C10—C9—C8	122.03 (15)	N3—C22—C21	122.97 (18)
C10—C9—Cl1	118.91 (12)	N3—C22—H22	118.5
C8—C9—Cl1	119.06 (13)	C21—C22—H22	118.5
C9—C10—C11	118.52 (15)	C4—N1—N2	103.89 (12)
C9—C10—H10	120.7	C17—N2—N1	110.81 (13)
C11—C10—H10	120.7	C17—N2—C18	127.39 (13)
C10—C11—C12	120.31 (15)	N1—N2—C18	121.72 (13)
C10—C11—H11	119.8	C18—N3—C22	117.54 (16)
C12—C11—H11	119.8	C13—O2—C14	117.31 (16)
C11—C12—C7	120.83 (15)	C2—O3—H3	109.5
C11—C12—H12	119.6	C17—O4—H4	109.5
C7—C12—H12	119.6		
			
C13—C1—C2—O3	70.80 (16)	C10—C11—C12—C7	−0.9 (3)
C6—C1—C2—O3	−53.11 (16)	C8—C7—C12—C11	1.0 (2)
C13—C1—C2—C16	−49.95 (17)	C6—C7—C12—C11	−176.00 (15)
C6—C1—C2—C16	−173.86 (13)	C6—C1—C13—O1	54.3 (2)
C13—C1—C2—C3	−171.33 (13)	C2—C1—C13—O1	−71.5 (2)
C6—C1—C2—C3	64.75 (16)	C6—C1—C13—O2	−125.83 (14)
O3—C2—C3—C4	66.92 (16)	C2—C1—C13—O2	108.41 (15)
C16—C2—C3—C4	−171.08 (13)	C4—C5—C17—O4	179.84 (17)
C1—C2—C3—C4	−49.14 (17)	C6—C5—C17—O4	2.2 (3)
C2—C3—C4—N1	−160.33 (14)	C4—C5—C17—N2	0.32 (17)
C2—C3—C4—C5	21.3 (2)	C6—C5—C17—N2	−177.30 (15)
N1—C4—C5—C17	−0.35 (18)	N3—C18—C19—C20	0.6 (3)
C3—C4—C5—C17	178.15 (14)	N2—C18—C19—C20	−179.03 (15)
N1—C4—C5—C6	177.45 (14)	C18—C19—C20—C21	−0.2 (3)
C3—C4—C5—C6	−4.0 (2)	C19—C20—C21—C22	−0.5 (3)
C17—C5—C6—C7	−44.3 (2)	C20—C21—C22—N3	0.9 (3)
C4—C5—C6—C7	138.48 (15)	C5—C4—N1—N2	0.23 (17)
C17—C5—C6—C1	−167.28 (16)	C3—C4—N1—N2	−178.26 (13)
C4—C5—C6—C1	15.5 (2)	O4—C17—N2—N1	−179.77 (14)
C13—C1—C6—C5	−170.09 (12)	C5—C17—N2—N1	−0.21 (18)
C2—C1—C6—C5	−45.43 (16)	O4—C17—N2—C18	3.5 (3)
C13—C1—C6—C7	64.71 (16)	C5—C17—N2—C18	−176.92 (14)
C2—C1—C6—C7	−170.63 (12)	C4—N1—N2—C17	−0.01 (16)
C5—C6—C7—C8	142.41 (15)	C4—N1—N2—C18	176.92 (13)
C1—C6—C7—C8	−95.41 (17)	N3—C18—N2—C17	5.5 (2)
C5—C6—C7—C12	−40.7 (2)	C19—C18—N2—C17	−174.84 (15)
C1—C6—C7—C12	81.54 (17)	N3—C18—N2—N1	−170.91 (13)
C12—C7—C8—C9	−0.5 (2)	C19—C18—N2—N1	8.8 (2)
C6—C7—C8—C9	176.50 (15)	C19—C18—N3—C22	−0.3 (3)
C7—C8—C9—C10	−0.1 (3)	N2—C18—N3—C22	179.38 (15)
C7—C8—C9—Cl1	−179.58 (12)	C21—C22—N3—C18	−0.5 (3)
C8—C9—C10—C11	0.2 (3)	O1—C13—O2—C14	3.9 (3)
Cl1—C9—C10—C11	179.72 (13)	C1—C13—O2—C14	−175.97 (16)
C9—C10—C11—C12	0.3 (3)	C15—C14—O2—C13	−79.9 (3)

### Hydrogen-bond geometry (Å, °)

**Table:**

*D*—H···*A*	*D*—H	H···*A*	*D*···*A*	*D*—H···*A*
O4—H4···N3	0.82	1.90	2.604 (3)	143
O3—H3···N1^i^	0.82	2.10	2.920 (2)	176
C11—H11···O3^ii^	0.93	2.58	3.418 (3)	151
C16—H16C···O4^iii^	0.96	2.57	3.397 (3)	144

Symmetry codes: (i) −*x*, −*y*, −*z*+1; (ii) *x*+1, *y*, *z*; (iii) *x*−1, *y*−1, *z*.
